# Wireless High Rotational Speed Assessment by Exploiting an RF Sensor Tag System and Equivalent-Time Reconstruction

**DOI:** 10.3390/s26092834

**Published:** 2026-05-01

**Authors:** Armin Gharibi, Filippo Costa, Simone Genovesi

**Affiliations:** Department of Information Engineering, University of Pisa, 56123 Pisa, Italy; filippo.costa@unipi.it (F.C.); simone.genovesi@unipi.it (S.G.)

**Keywords:** wireless sensing, rotational speed measurement, RF impedance sensing, equivalent-time sampling, nonuniform sampling, phase-domain reconstruction, sparse sampling

## Abstract

Rotational speed monitoring is essential in many industrial and electromechanical systems. This paper presents a rotational speed measurement method based on a wireless impedance sensing system leveraging the radio-frequency coupling between a passive resonant tag and a coplanar waveguide (CPW) probe. The sensing mechanism exploits periodic variations in the real part of the probe impedance caused by the relative alignment between the rotating tag and the stationary probe. While the impedance signal is inherently periodic, the usable speed range of sampling-based measurement systems is fundamentally constrained by their acquisition rate. To overcome this limitation without requiring higher-rate instrumentation, an equivalent-time sampling (ETS) reconstruction approach is proposed. Sparse and nonuniform impedance samples collected over multiple revolutions are mapped into an equivalent phase domain and combined to reconstruct the waveform associated with a single rotation period. The method is reader-agnostic in principle, as it only requires time-stamped monitoring of a periodic RF observable at a selected frequency; however, experimental validation in this work is performed using a vector network analyzer (VNA). Experimental results obtained on a rotating platform with speeds ranging from 150 RPM to 4000 RPM demonstrate that the proposed method reduces the mean relative estimation error to below 5% across the full range, compared to errors exceeding 70% for conventional peak-based estimation above 1000 RPM. These results highlight the effectiveness of the ETS approach in extending the operational range of RF impedance-based rotational sensing under severe undersampling conditions. The proposed framework is generalizable to other periodic RF sensing configurations where signal periodicity can be exploited across multiple acquisition cycles.

## 1. Introduction

Rotational speed monitoring is a key requirement in many industrial and electromechanical systems, including electric motors, rotating machinery, robotics, and automated manufacturing equipment. Accurate measurement of rotational motion supports condition monitoring, fault detection, and closed-loop control, making rotational sensing an important function in both industrial automation and machine diagnostics [1].

A wide range of sensing technologies has been developed for rotational speed measurement, each with specific advantages and limitations. Optical techniques, including encoders and vision-based systems, can provide high accuracy and resolution, but they require direct line of sight and are sensitive to dust, oil, and debris. Fiber-optic sensors offer immunity to electromagnetic interference, although they often require precise optical alignment and dedicated instrumentation [2]. Magnetic sensors, including giant magnetoresistance (GMR)-based devices, are widely used in industrial environments because of their robustness, but they generally require dedicated magnetic targets and may be affected by electromagnetic interference [3]. In addition, conventional encoder-based solutions can provide very high accuracy but typically require direct mechanical coupling and precise installation [4].

Other approaches have also been investigated. Electrostatic sensors can estimate rotational speed from charge variations generated by rotating components [5], while surface acoustic wave (SAW) sensors provide passive sensing capability with high sensitivity [6]. Radar-based techniques, including millimeter-wave systems, enable non-contact rotational sensing through Doppler-based measurements [7], and optical Doppler-based methods have also been proposed for ultra-low-speed detection [8]. Furthermore, RFID-based techniques have been explored for monitoring rotating machinery and detecting mechanical anomalies such as eccentricity [9]. Despite their advantages, many of these solutions require complex hardware, relatively high power consumption, or specially designed targets.

Wireless and passive sensing approaches have attracted increasing attention as alternatives to conventional wired sensors. Early RF-based methods demonstrated high-speed rotational sensing through wireless signal transmission [10], while passive resonant schemes have been used to generate periodic responses associated with the rotating target [11]. Microwave techniques have also been proposed for contactless rotational monitoring. For example, Jha et al. [12] reported a microwave sensor for combined rotation and proximity detection, and Bai et al. [13] demonstrated rotational speed measurement using a near-field open-ended waveguide probe.

Among passive RF solutions, chipless radio-frequency identification (RFID) technology has emerged as a promising platform for low-cost sensing. Chipless RFID tags are based on resonant electromagnetic structures and do not require integrated circuits, which makes them attractive for passive and inexpensive sensing applications. Resonator-based microwave encoders have been proposed for angular velocity sensing and chipless RFID systems [14], while additional developments have demonstrated rotation direction detection and improved encoding strategies [15]. Chipless RFID structures have also been used for angular rotation monitoring [16], and more recent developments include battery-free wireless sensing concepts for rotational and joint monitoring [17].

More recently, Gharibi et al. [18] introduced a wireless sensing configuration based on a passive resonant tag and a coplanar waveguide (CPW) probe. In that system, the periodic alignment between the rotating tag and the stationary probe produces measurable variations in the real part of the probe impedance. Besides rotational information, the same sensing platform can also provide information about angular position, alignment, and distance variation.

A key advantage of this RF impedance-based sensing approach is its inherent multi-functionality. By appropriately selecting and processing the measured RF response, the same hardware configuration can be adapted to different sensing tasks. In addition to angular sensing, the platform has been successfully applied to robotic hand localization and object recognition in recent works [19,20]. These studies highlight the versatility of passive RF sensing systems, where multiple functionalities can be achieved without modifying the hardware, but rather by exploiting different features of the measured signal.

These characteristics make RF impedance sensing particularly attractive for industrial and robotic scenarios, since it is compact, passive, low cost, and capable of operating behind thin non-metallic barriers where optical methods may be difficult to deploy.

It is important to emphasize that the novelty of the present work does not primarily lie in the sensing hardware itself, which builds upon the previously reported CPW probe and passive tag configuration in Ref. [18]. Instead, the main contribution of this work is a signal-processing framework based on equivalent-time reconstruction, which enables reliable rotational speed estimation under severe undersampling conditions.

A major limitation of RF impedance-based sensing arises from the measurement instrument. When a vector network analyzer (VNA) is used to monitor impedance variations, the available acquisition rate may be insufficient to capture the rapid signal changes that occur at high rotational speeds. As the rotation speed increases, impedance peaks associated with tag–probe alignment may be sparsely sampled or completely missed, causing conventional peak-based speed estimation to become unreliable.

To address this limitation, this work introduces an equivalent-time reconstruction method for RF impedance-based rotational sensing. The proposed approach combines sparse and nonuniform impedance samples collected over multiple revolutions, maps them into an equivalent phase domain, and reconstructs the impedance waveform associated with a single rotation period. In this way, reliable rotational speed estimation becomes possible even when individual alignment events are not directly observable in the time domain.

An important advantage of the proposed approach is that it is not inherently tied to laboratory-grade instrumentation. Since the method only requires time-stamped monitoring of a periodic RF observable at a selected operating frequency, the measurement front-end can, in principle, be implemented using low-cost portable readers or simplified single-frequency RF interrogation circuits. This is consistent with recent developments in compact reflective-mode microwave sensing systems and low-cost vector network analyzer architectures [21,22]. However, in the present work, experimental validation is carried out using a vector network analyzer.

In practical implementations, the proposed method is intended for quasi real-time operation. The main latency is not associated with the numerical processing itself, which consists of phase folding, binning, interpolation, and evaluation of a finite set of candidate periods, but rather with the time required to collect a sufficient number of samples for reliable reconstruction. Similar timing constraints have also been discussed in RF/RFID-based sensing systems for rotating targets, where the achievable response time is governed primarily by the sensing protocol and data accumulation requirements rather than by the computational cost alone [9,23].

Consequently, the proposed method provides a scalable and cost-effective solution for rotational monitoring in practical industrial scenarios, while significantly extending the usable operating range of RF impedance-based sensing beyond the limits imposed by conventional sampling constraints.

The main contributions of this work are summarized as follows:

- A wireless rotational speed sensing system based on RF impedance variation using a passive resonant tag and a CPW probe.
- A rotational sensing mechanism based on periodic impedance modulation caused by tag–probe alignment.
- An equivalent-time reconstruction method for robust speed estimation under sparse sampling conditions.
- Experimental validation over a rotational speed range from 150 RPM to 4000 RPM.
- A comparative assessment showing that the proposed method extends the usable operating range of RF impedance-based rotational sensing beyond conventional peak-based estimation.

Unlike conventional ETS applications, which typically assume uniform or band-limited signals with known periodicity, the proposed approach addresses nonuniform impedance sampling, unknown rotational periods, and non-bandlimited signals arising from RF sensing.

Table 1 compares the proposed approach with representative rotational speed sensing technologies reported in the literature.

## 2. Sensor Principle and Operating Mechanism

The proposed sensing approach exploits the electromagnetic interaction between a rotating passive resonant tag (i.e., without an integrated circuit) and a stationary coplanar waveguide (CPW) probe connected to a vector network analyzer (VNA). As the tag passes through the near field of the probe, the coupling between the two structures varies, producing a time-varying impedance response. Since this interaction occurs once per revolution, the rotational motion can be inferred from the resulting periodic impedance variation.

The sensing element is a chipless passive resonant tag implemented as a planar electromagnetic structure. Unlike conventional RFID tags, chipless tags do not contain integrated circuits and interact with the incident RF field solely through their resonant behavior. In the proposed configuration, the tag is mounted on a rotating disk and periodically passes through the sensing region beneath the probe. When the tag approaches the probe, the electromagnetic interaction modifies the impedance observed at the antenna port.

**Figure 1:**
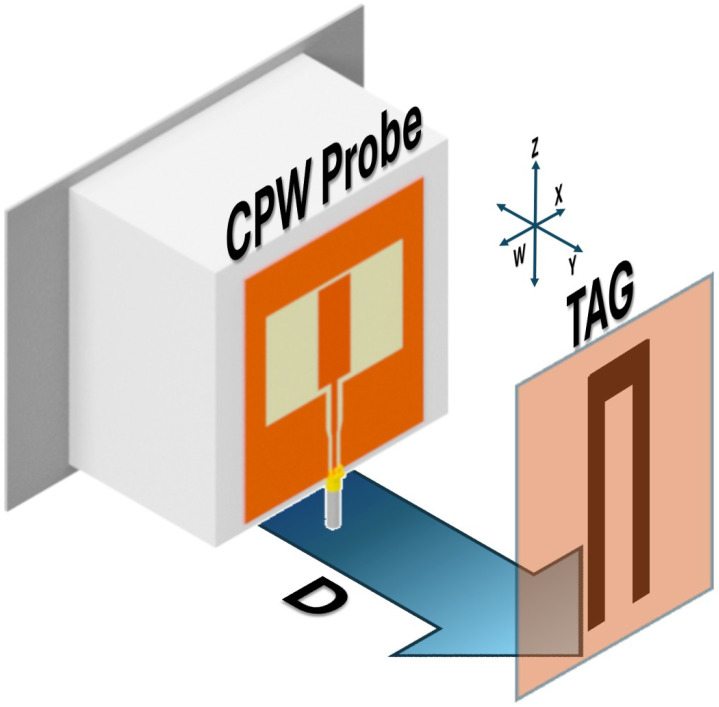
Sensing configuration: a stationary CPW probe is positioned in front of a rotating chipless resonant tag. The parameter *D* represents the separation distance between the two structures.

The sensing geometry is illustrated in Figure 1, where *D* denotes the separation distance between the probe and the tag. Both the distance and the relative alignment influence the coupling strength. In this work, the separation distance was set to 10 mm. Previous studies have shown that the tag can be reliably detected over a wider range of distances, from 0 to 60 mm [18].

The tag was fabricated on a flexible EVA foam substrate using conductive ink. Its geometry consists of a U-shaped structure with an arm length of 63 mm and a spacing of 10 mm between the arms. The probe is implemented as a CPW structure connected to a VNA, which monitors the impedance at a selected frequency.

The measured impedance is expressed as

(1) Z=R+jX,

where *R* and *X* denote the real and imaginary components, respectively. In this work, the real part *R* is used for rotational sensing due to its higher sensitivity to tag–probe alignment.

As the disk rotates, the relative position between the tag and the probe changes continuously. When the resonant element aligns with the probe, the electromagnetic coupling is maximized, resulting in a distinct perturbation of the probe impedance. As the tag moves away, the coupling decreases and the impedance returns toward its baseline value. This periodic interaction produces a sequence of impedance peaks, each corresponding to a single revolution of the rotating structure.

The resulting time-domain impedance response can be expressed as

(2) $$R\left(t\right)=R_{0}+\Delta R\left(t\right),$$

where $R_{0}$ is the baseline real part of the impedance and ΔR(t) represents the variation induced by the rotating tag. Under adequate sampling conditions, each alignment event produces a distinguishable peak in R(t), and the time interval between consecutive peaks corresponds to the rotational period.

Let $T_{r}$ denote the time interval between two successive impedance peaks. The rotational speed can then be expressed as

(3) $$RPM=\frac{60}{T_{r}}.$$

At low rotational speeds, the impedance peaks are clearly resolved and the rotational speed can be directly estimated. However, as the rotational speed increases, the acquisition rate of the VNA becomes insufficient to capture all peaks, leading to sparse sampling and potential loss of peak information. To address this limitation, Section 4 introduces an equivalent-time reconstruction approach, which enables accurate rotational speed estimation even under undersampled conditions.

## 3. Measurement System and Experimental Setup

**Figure 2:**
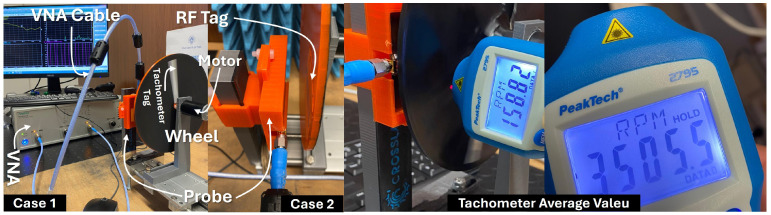
Experimental setup used for the rotational speed measurements.

The experimental setup (Figure 2) was designed to evaluate the proposed RF impedance-based rotational sensing approach under controlled laboratory conditions. The system consists of a chipless passive resonant tag mounted on a rotating platform and a stationary CPW probe connected to a vector network analyzer (VNA).

The RF measurement system is based on a CPW probe connected to an Anritsu MS46122B vector network analyzer (Anritsu Corporation, Atsugi, Japan) via a coaxial cable. The VNA monitors the impedance response of the probe while the tag rotates in front of it. Prior to the rotation experiments, the frequency response of the tag–probe pair was characterized to identify a suitable sensing region. A resonance was observed around 1.79 GHz in the $S_{11}$ response. Based on this analysis, a frequency region around 1.94 GHz was identified as highly sensitive to tag–probe alignment, providing strong impedance variation under ideal conditions.

The chipless resonant tag was implemented as a U-shaped conductive structure printed with conductive ink on an EVA foam substrate. The tag was mounted on a plastic wheel with a thickness of approximately 5 mm, attached to a rotating shaft driven by a DC motor capable of speeds up to 4000 RPM. The rotational speed was controlled using an Arduino-based controller, while an optical tachometer (PeakTech 2795, PeakTech GmbH, Ahrensburg, Germany), was used as a reference measurement.

The rotational speed remained approximately constant during each acquisition window, with only minor fluctuations observed from the reference tachometer measurements, ensuring the validity of the ETS assumption of quasi-stationary rotational speed.

Impedance measurements were performed using the VNA operating in frequency-sweep mode. Each measurement consisted of a sweep from 1.5 to 2.0GHz with 59 frequency points. The real and imaginary parts of the impedance were recorded together with time stamps, resulting in a nonuniformly sampled time series.

**Figure 3:**
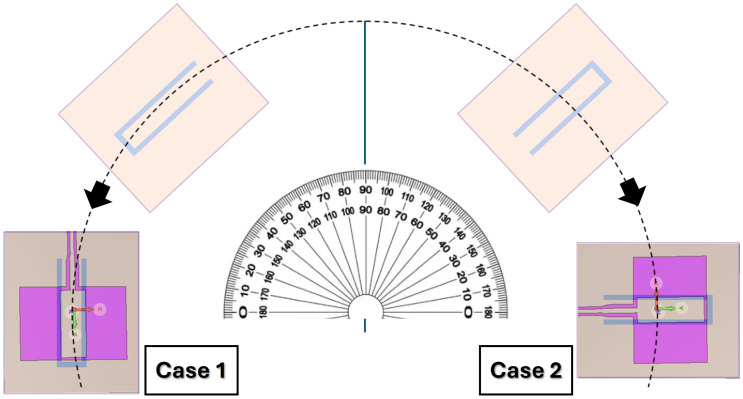
Alignment configurations between the CPW probe and the rotating chipless resonant tag.

Although frequencies around 1.94 GHz exhibit high sensitivity under ideal alignment conditions (Figure 3), a systematic evaluation across the dataset was conducted to identify the most robust operating frequency for rotational speed estimation. Candidate frequencies in the range of approximately 1.93–1.95GHz were analyzed. Among these, the frequency at approximately 1.95GHz was selected for subsequent processing, as it provided the most stable and accurate results, particularly under sparse sampling conditions. This observation indicates that the frequency maximizing instantaneous sensitivity is not necessarily the one that yields the best estimation performance.

For each rotational speed condition, approximately 1000 samples were acquired over a duration of 25–30s, corresponding to an effective acquisition rate of approximately 33–40Hz. Each sample is represented as $\left(t_{i},z_{i}\right)$, where $t_{i}$ is the acquisition time and $z_{i}=ℜ\left\{Z\left(t_{i}\right)\right\}$. The resulting time series was used as input for both the peak-based method and the equivalent-time reconstruction approach described in the following sections.

This acquisition duration was selected as a conservative setting to ensure robust reconstruction across the full investigated speed range, including the most severely undersampled cases. In practice, the observation window also determines the response latency of the ETS method, since the estimation accuracy depends on the number of accumulated samples. Therefore, $T_{\mathrm{obs}}$ represents a key tradeoff between robustness and update speed. The main experimental data acquisition parameters used in this study are summarized in Table 2.

## 4. Rotational Speed Estimation from Impedance Peaks

### 4.1. Background and Method Selection

Estimating rotational speed from measured signals is a well-established problem in condition monitoring and sensing systems. Conventional approaches typically rely on time-domain, frequency-domain, or model-based techniques to extract periodic features associated with rotational motion. For instance, autoregressive (AR) models and other time-series methods attempt to predict signal evolution, but they often exhibit limited accuracy in fast rotating environments due to rapidly varying dynamics and multipath effects [25]. Spectral methods such as the Lomb–Scargle periodogram and autocorrelation-based techniques are commonly employed to detect periodicity, particularly under nonuniform sampling conditions. However, these approaches generally assume smooth or sinusoidal signal structures and may not be well suited for signals characterized by impulsive or sparse features [26].

In parallel, time–frequency analysis techniques, including short-time Fourier transform (STFT), Wigner–Ville distribution (WVD), and wavelet transforms, as well as compressive sensing (CS) approaches, have been widely used in rotating machinery diagnostics. While these methods provide powerful tools for signal reconstruction and feature extraction, they typically require relatively dense sampling and may involve significant computational complexity [27]. Furthermore, machine learning-based approaches have recently been proposed for rotational analysis and fault diagnosis, but they require large training datasets and are not always suitable for analytical modeling or real-time implementation. The main characteristics of these analytical methods are summarized in Table 3.

In the context of RF impedance-based sensing, the measured signal is characterized by sparse, nonuniform samples and impulsive peak-like behavior rather than continuous waveforms. As a result, many of the aforementioned approaches are either not directly applicable or lead to reduced estimation accuracy. This motivates the need for a dedicated strategy tailored to the characteristics of the measured signal.

Based on the above considerations, conventional methods either rely on assumptions that are not satisfied by the RF impedance signal such as sinusoidal behavior or dense sampling or require computationally intensive processing.

As a result, a peak-based method is adopted as a baseline approach. This choice is motivated by the physical nature of the sensing mechanism, where each rotation produces a distinct impedance peak corresponding to the tag–probe alignment event. Under adequate sampling conditions, the time interval between successive peaks directly provides the rotation period, making peak detection a natural and widely used reference method for rotational speed estimation.

However, due to the sparse and asynchronous nature of the measurements, this method exhibits limited accuracy, particularly at high rotational speeds where peaks may be partially sampled or completely missed.

To overcome these limitations, an equivalent-time sampling (ETS) based reconstruction approach is proposed. By exploiting the inherent periodicity of the rotating system, ETS enables the reconstruction of a high-resolution representation of the impedance signal from sparse, nonuniform samples, allowing for accurate and robust rotational speed estimation across a wide operating range.

### 4.2. Peak Detection Method

The rotational speed can be estimated directly from the time-domain impedance signal by identifying periodic alignment events. At low rotational speeds, the impedance signal exhibits clearly identifiable peaks corresponding to successive tag–probe alignment events, as described in Section 2. These peaks are used as reference points for estimating the rotation period.

To improve robustness against measurement noise, the impedance signal is preprocessed using a median filter followed by a moving-average filter. This step suppresses small fluctuations and enhances the prominence of the alignment-induced peaks. Candidate peaks are then detected using a prominence-based criterion, ensuring that only significant local maxima are retained.

**Figure 4:**
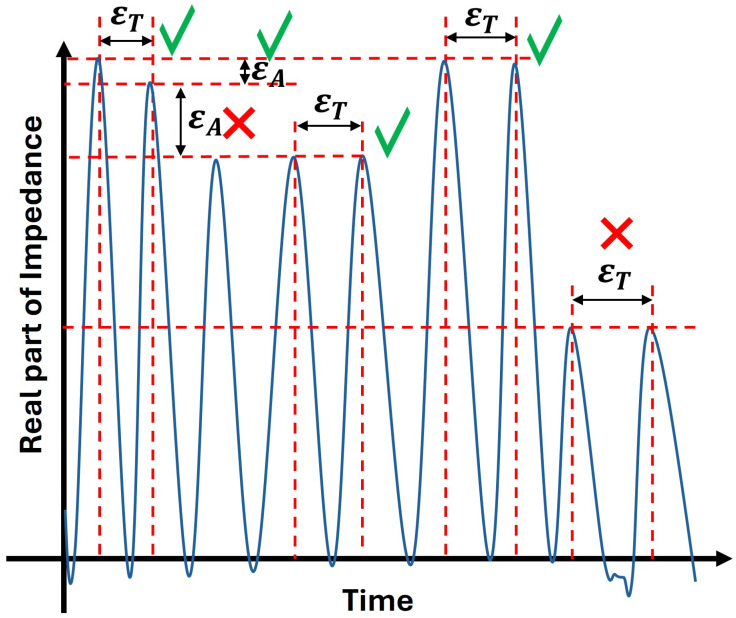
Illustration of the peak selection criteria used for reliable rotation period estimation from the real part of the impedance signal. The temporal spacing between consecutive peaks, denoted as $\varepsilon_{T}$, is used to estimate the rotational period, while the peak amplitude difference, $\varepsilon_{A}$, reflects variations in signal strength. Valid peak pairs (√) satisfy both temporal consistency and sufficient amplitude similarity, ensuring accurate period estimation. In contrast, peak pairs marked with (×) are rejected due to either large amplitude deviations or inconsistent temporal spacing, which can lead to erroneous rotational speed estimation.

Figure 4 illustrates the peak selection criteria. A valid pair corresponds to two consecutive alignment events and must satisfy both temporal and amplitude consistency. This selection process ensures that only peaks corresponding to true tag–probe alignment events are used, preventing incorrect period estimation caused by spurious or partially sampled peaks.

To estimate the rotation period, an adaptive peak matching procedure is applied. An approximate nominal rotational speed, denoted as $RPM_{ref}$, is used to define a reference rotation period

(4) $$T_{r}^{ref}=\frac{60}{RPM_{ref}}.$$

Here, $RPM_{ref}$ is a nominal reference rotational speed used solely to guide the peak matching process and does not need to be accurate.

Detected peaks are then paired based on two conditions. First, the temporal spacing between peaks must be consistent with the nominal rotation period,

(5) $$\left|\left(t_{j}-t_{i}\right)-T_{r}^{ref}\right|<\epsilon_{T}T_{r}^{ref}.$$

Second, the amplitudes of the peaks must be comparable,

(6) $$\frac{|p_{j}-p_{i}|}{\max(|p_{i}|,|p_{j}|)}<\epsilon_{A}.$$

An adaptive strategy is employed in which strict tolerance values are initially applied. If no valid peak pairs are found, the tolerances are progressively relaxed. When multiple valid peak pairs are available, all corresponding time intervals are retained (Figure 4).

The parameters used in the peak detection and matching procedure are summarized in Table 4. These values were selected empirically to ensure robust performance across the investigated rotational speed range.

### 4.3. Rotation Period Estimation

The rotation period is estimated from the time difference between consecutive matched peaks,

(7) $$T_{r}=t_{i+1}-t_{i}.$$

When multiple estimates are available, the final rotation period is computed as the median of all valid intervals, reducing the influence of outliers. The rotational speed is then obtained from the estimated rotation period using the relationship defined in Equation (3).

The performance of the peak-based method is influenced by the choice of the tolerance parameters $\epsilon_{T}$ and $\epsilon_{A}$, as well as the peak detection threshold. These parameters were selected empirically to achieve a compromise between accuracy and robustness. In particular, the initial tolerance values were chosen to minimize estimation error at low rotational speeds, where peaks are clearly resolved, while the relaxed values enable the selection of the best available peak candidates under sparse sampling conditions at higher speeds.

Although the estimation accuracy depends on these parameters, the use of adaptive threshold relaxation reduces sensitivity to their exact values and improves robustness across different operating conditions. Nevertheless, even with careful parameter tuning, the method remains fundamentally limited when the number of samples per revolution becomes very low, as valid peaks may be partially sampled or completely missed.

### 4.4. Algorithm Characteristics and Limitations

The peak-based method relies on the availability of sufficient samples within each rotation period. As the rotational speed increases, the number of samples per revolution decreases due to the limited acquisition rate of the VNA.

The reduction in sampling density as a function of rotational speed is summarized in Table 5. This illustrates the fundamental limitation of peak-based estimation at high speeds.

Under sparse sampling conditions, impedance peaks may be partially captured or completely missed, leading to unreliable estimation.

It is important to note that the nominal speed $RPM_{ref}$ is used only as a guiding parameter for peak matching. The method remains sensitive to its accuracy and may fail when the signal is severely undersampled.

These limitations motivate the use of the equivalent-time reconstruction approach described in the next section.

Table 6 shows the performance of the peak-based estimator for the two measurement configurations. At low rotational speed, the method provides acceptable accuracy. However, as the rotational speed increases, the estimation error grows rapidly in both cases due to missed or partially sampled impedance peaks. Above approximately 1000 RPM, the peak-based method becomes unreliable, with errors exceeding 1000 RPM in several operating conditions. This quantitative result confirms the limitations imposed by sparse sampling and motivates the equivalent-time reconstruction approach introduced in the next section.

## 5. Equivalent-Time Reconstruction Method

To overcome the limitations of the peak-based method described in the previous section, an equivalent-time reconstruction (ETS) approach is introduced for robust rotational speed estimation under sparse sampling conditions.

### 5.1. Sparse Sampling at High Rotational Speeds

As the rotational speed increases, the duration of a single revolution decreases while the acquisition rate of the vector network analyzer (VNA) remains fixed. Consequently, the number of samples captured within each rotation period rapidly decreases and may become smaller than one at high speeds.

Under these conditions, impedance peaks associated with tag–probe alignment are not consistently sampled, making direct peak-based estimation unreliable. However, the impedance signal remains inherently periodic, since the electromagnetic interaction between the rotating tag and the probe repeats once per revolution. This periodicity enables reconstruction techniques that combine measurements collected over multiple rotations.

### 5.2. Equivalent-Time Reconstruction Approach

Equivalent-time sampling (ETS) techniques have been widely used to reconstruct periodic signals from measurements acquired at sampling rates below the Nyquist limit. These approaches exploit signal periodicity to combine samples collected over multiple cycles into a single equivalent period, effectively increasing the sampling resolution.

Prior works have demonstrated ETS in biomedical signal processing and high-frequency measurement systems [28,29,30]. However, most existing approaches assume band-limited signals with known periodicity and often rely on uniformly sampled data [31].

In contrast, the present work addresses a more challenging scenario characterized by:

- Nonuniform and sparse time sampling due to VNA sweep operation.
- Unknown rotational period that must be estimated from data.
- Non-bandlimited, impulsive impedance signals with peak-like behavior.

Under these conditions, classical ETS formulations cannot be directly applied. The proposed method therefore estimates the rotational period by exploiting phase consistency across multiple revolutions without requiring prior knowledge of the signal frequency or strict band-limited assumptions.

### 5.3. Signal Preprocessing

Prior to reconstruction, the impedance signal is preprocessed to improve robustness. The real part of the impedance at the selected sensing frequency is extracted as

(8) $$z\left(t_{i}\right)=ℜ\left\{Z\left(f_{s},t_{i}\right)\right\},$$

and normalized as

(9) $$z_{\mathrm{norm}}\left(t_{i}\right)=\frac{z\left(t_{i}\right)-\mathrm{median}\left(z\left(t\right)\right)}{\max|z(t)|}.$$

This preprocessing reduces baseline variations and emphasizes the periodic component associated with the tag–probe alignment events.

### 5.4. Phase-Domain Reconstruction

Let $\left(t_{i},z_{i}\right)$ denote the measured samples, where $t_{i}$ is the acquisition time and $z_{i}=ℜ\left\{Z\left(t_{i}\right)\right\}$. Although these samples are nonuniformly distributed in time, they can be mapped into an equivalent phase domain.

For a given candidate rotation period $T_{k}$, each sample is assigned a normalized phase

(10) $$\phi_{i}=\frac{t_{i}\;\mathrm{mod}\;T_{k}}{T_{k}},$$

which folds all samples acquired over multiple revolutions into the interval ϕ∈[0,1).

The phase interval is divided into $N_{b}$ bins, and samples within each bin are aggregated to obtain representative values. In this work, $N_{b}=24$ was selected empirically as a compromise between phase resolution and reconstruction robustness. Smaller values result in coarse phase representation, while larger values increase sparsity and sensitivity to noise. This value provided adequate phase resolution while maintaining sufficient average sample support per bin over the investigated operating range, thereby reducing the occurrence of poorly populated bins under sparse sampling conditions.

For each bin, a representative value is computed using local averaging or median aggregation. The resulting support points are then used to reconstruct the impedance waveform over one rotation period using periodic interpolation.

### 5.5. Rotation Period Estimation via Search

The rotational period is estimated through a search over a predefined range of candidate rotational speeds. For each candidate value $RPM_{k}$, the corresponding period is

(11) $$T_{k}=\frac{60}{RPM_{k}}.$$

For each $T_{k}$, phase-domain reconstruction is performed as described above. The quality of reconstruction is quantified using a consistency score that evaluates how well the measured samples align into a coherent periodic waveform.

Let ${\hat{z}}_{k}\left(\phi \right)$ denote the reconstructed waveform associated with $T_{k}$. The consistency score is defined as

(12) $$S_{k}=\rho_{k}\frac{\mathrm{Var}\;\left({\hat{z}}_{k}\right)}{{\mathrm{MSE}}_{k}+\varepsilon },$$

where $\rho_{k}$ is the occupancy ratio of valid phase bins,

(13) $$\rho_{k}=\frac{N_{\mathrm{valid}}}{N_{b}},$$

and ${\mathrm{MSE}}_{k}$ is the mean squared error between measured samples and reconstructed values,

(14) $${\mathrm{MSE}}_{k}=\frac{1}{N}\sum_{i=1}^{N}{\left(z_{i}-{\hat{z}}_{k}\left(\phi_{i}\right)\right)}^{2}.$$

Here, $N_{b}$ is the number of phase bins, $N_{\mathrm{valid}}$ is the number of bins containing sufficient samples, and ε is a small constant introduced for numerical stability. This formulation favors candidate periods that provide strong waveform contrast, good phase coverage, and low reconstruction error.

The optimal rotation period is selected as

(15) $$T_{r}=\arg\max_{T_{k}}S_{k},$$

and the rotational speed is finally obtained as

(16) $$RPM=\frac{60}{T_{r}}.$$

The overall procedure of the proposed ETS-based rotational speed estimation method is summarized in Algorithm 1.
**Algorithm 1** Equivalent-Time Reconstruction (ETS) for Rotational Speed Estimation**Require:** 
Nonuniform samples $\left(t_{i},z_{i}\right)$, candidate speed range $\left[RPM_{\min},RPM_{\max}\right]$, number of phase bins $N_{b}$**Ensure:** 
Estimated rotational speed RPM1:Extract impedance signal:$$z_{i}\leftarrow ℜ\left\{Z\left(f_{s},t_{i}\right)\right\}$$2:Normalize signal:$$z_{i}\leftarrow \frac{z_{i}-\mathrm{median}\left(z\right)}{\max|z|}$$3:**for** each candidate $RPM_{k}$ in $\left[RPM_{\min},RPM_{\max}\right]$ **do**4:       Compute candidate period:$$T_{k}=\frac{60}{RPM_{k}}$$5:       Map samples to phase domain:$$\phi_{i}=\frac{t_{i}\;\mathrm{mod}\;T_{k}}{T_{k}}$$6:       Divide ϕ∈[0,1) into $N_{b}$ bins7:       Compute representative value per bin8:       Reconstruct periodic waveform ${\hat{z}}_{k}\left(\phi \right)$9:       Evaluate consistency score $S_{k}$ using Equations (12)–(14)10:**end for**11:Select optimal period:$$T_{r}=\arg\max S_{k}$$12:Compute rotational speed:$$RPM=\frac{60}{T_{r}}$$

### 5.6. Stability Considerations

The ETS reconstruction assumes that the rotational speed remains approximately constant over the observation window. Let ΔΩ denote the variation in rotational speed during $T_{\mathrm{obs}}$. The resulting phase drift can be approximated as

(17) $$\Delta \phi \approx \frac{\Delta \Omega }{\Omega }\;\cdot \;\frac{T_{\mathrm{obs}}}{T_{r}}.$$

If Δϕ≪1, samples remain phase-consistent and can be combined effectively. Otherwise, phase misalignment leads to waveform blurring and reduced estimation accuracy.

### 5.7. General Sampling Rate Analysis

The ETS method relies on accumulating samples over multiple revolutions rather than capturing multiple samples within a single rotation. The total number of samples collected during the observation window is

(18) $$N_{s}=f_{\mathrm{acq}}T_{\mathrm{obs}},$$

where $f_{\mathrm{acq}}$ is the acquisition rate. A useful indicator of reconstruction quality is the average number of samples per phase bin,

(19) $$\Gamma =\frac{N_{s}}{N_{b}}.$$

A larger Γ improves phase coverage and reconstruction robustness. Even when the number of samples per revolution is less than one, ETS remains effective by combining measurements across multiple rotations.

The time evolution of phase coverage can be expressed as

(20) $$\Gamma \left(t\right)=\frac{f_{\mathrm{acq}}\;t}{N_{b}}.$$

After an initial accumulation phase, Γ becomes sufficiently large for reliable estimation, allowing continuous updates of the rotational speed in a quasi real-time manner.

Among the algorithmic parameters, the observation window $T_{\mathrm{obs}}$ is particularly important because it directly affects both estimation accuracy and latency. A longer observation window increases the total number of available samples and improves the average sample support per phase bin, which generally leads to more stable reconstruction and more accurate period estimation. On the other hand, a shorter observation window reduces response time but may lead to insufficient phase coverage, especially at low rotational speeds or under strongly sparse sampling conditions. For this reason, $T_{\mathrm{obs}}$ should be selected according to the requirements of the target application, balancing robustness against update speed.

### 5.8. Computational Cost and Quasi Real-Time Operation

From a computational point of view, the ETS method is relatively lightweight. For each candidate rotational speed, the algorithm performs phase folding, phase-bin aggregation, interpolation, and evaluation of the consistency score. These operations scale approximately linearly with the number of acquired samples and the number of candidate periods considered in the search. As a result, the main limitation for real-time operation is not the numerical complexity of the algorithm, but the observation time required to accumulate a sufficient number of samples for reliable phase-domain coverage.

Accordingly, the proposed method is better described as a quasi real-time estimator. After a short initialization interval, newly acquired samples can be incorporated into a rolling buffer and the estimate can be continuously refreshed without requiring a complete restart of the acquisition process. Therefore, the effective latency is primarily governed by the chosen observation window $T_{\mathrm{obs}}$, which directly controls the tradeoff between update speed and reconstruction robustness.

## 6. Performance Evaluation

The performance of the proposed method was evaluated over a range of reference speeds from 150 RPM to 4000 RPM. The analysis first examines the influence of frequency selection and measurement configuration, and then presents detailed reconstruction and estimation results for the preferred configuration.

### 6.1. Frequency Selection and Configuration Analysis

To evaluate the influence of both sensing frequency and measurement configuration, experiments were conducted at three operating frequencies within the high-sensitivity region (approximately 1.93–1.95 GHz) for two different probe–tag alignment configurations, denoted as Case 1 and Case 2. To quantitatively compare the tested operating frequencies, the ETS estimation performance in terms of mean absolute and relative error is summarized in Table 7.

Although the lowest mean error is observed at 1.93 GHz, the selection of the operating frequency is guided not only by average accuracy but also by robustness at high rotational speeds, which is the primary objective of this study. In particular, 1.95 GHz exhibits more consistent tracking behavior and lower worst-case deviation at speeds above 3000 RPM, where sparse sampling effects become dominant.

**Figure 5:**
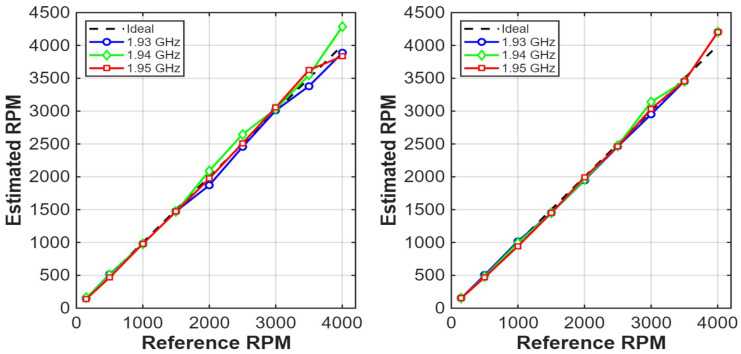
Comparison of ETS-estimated rotational speed at different sensing frequencies. (**Left**): Case 1. (**Right**): Case 2. The black dashed line indicates the ideal reference trend, while the colored curves correspond to the tested sensing frequencies.

This improved robustness is reflected in the reduced spread of estimation error at the upper end of the speed range, as observed in Figure 5. Therefore, despite the slightly lower mean error at 1.93 GHz, 1.95 GHz provides a more reliable performance under the most challenging operating conditions and is selected for the final evaluation.

Figure 5 compares the estimated rotational speed obtained at the three tested frequencies for both configurations. In both cases, all frequencies provide good agreement with the reference speed over a large portion of the investigated range. However, noticeable differences emerge at higher rotational speeds. In Case 1, the estimates at 1.94 GHz deviate more significantly from the ideal trend at the highest speed, while the results at 1.95 GHz remain closer to the reference. In Case 2, the three frequencies exhibit similar behavior over most of the range, but the estimates at 1.95 GHz show slightly more consistent tracking at the upper end of the speed range.

These observations indicate that the frequency maximizing instantaneous impedance contrast (around 1.94 GHz) is not necessarily optimal for rotational speed estimation. Instead, 1.95 GHz provides a better compromise between sensitivity and robustness under sparse sampling conditions, and is therefore adopted in the following analysis. This behavior also highlights the flexibility of the proposed system, where the operating frequency can be selected according to the desired tradeoff between average estimation accuracy and robustness under the most challenging sampling conditions.

To further assess the influence of the measurement configuration, Table 8 summarizes the ETS estimation results at the selected operating frequency for both cases.

Case 2 provides consistently better performance, particularly at high rotational speeds. This behavior can be explained by the characteristics of near-field electromagnetic coupling between the probe and the rotating tag. In such systems, sensing performance is not determined solely by the duration of the interaction, but rather by the strength and spatial distribution of the coupling, which depend on the relative geometry and orientation of the structures.

Although the tag passes in front of the probe over a shorter angular interval in Case 2, the probe–tag configuration likely produces stronger near-field coupling. This leads to more pronounced and higher-contrast impedance variations, with sharper and more distinguishable features in the measured signal. As a result, the phase-domain representation obtained after folding exhibits improved coverage, enhancing the stability of the ETS reconstruction and leading to more accurate rotational speed estimation under sparse sampling conditions. For this reason, Case 2 was selected as the representative configuration for the detailed analyses presented in the following sections.

**Figure 6:**
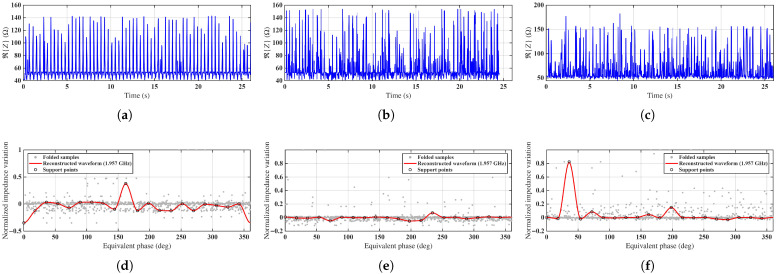
Representative impedance measurements and ETS reconstructions for Case 2 at approximately 1.95 GHz. (**a**–**c**) Raw impedance signals at 150 RPM, 2000 RPM, and 4000 RPM, respectively. (**d**–**f**) Corresponding ETS reconstructed waveforms at 150 RPM, 2000 RPM, and 4000 RPM.

Figure 6 presents representative results of the proposed method for Case 2 at three rotational speeds: 150 RPM, 2000 RPM, and 4000 RPM. The top row shows the raw time-domain impedance signals, while the bottom row illustrates the corresponding equivalent-time reconstructed waveforms.

At low rotational speed (150 RPM), the impedance signal exhibits clearly resolved and regularly spaced peaks corresponding to successive tag–probe alignment events. In this regime, both peak-based and ETS-based approaches are expected to perform reliably. The ETS reconstruction closely follows the underlying waveform and confirms the periodic structure of the signal.

At intermediate speed (2000 RPM), the number of samples per revolution is significantly reduced, and the raw signal becomes increasingly sparse. Individual peaks are no longer consistently captured, making direct peak detection unreliable. Nevertheless, the ETS method successfully reconstructs a smooth and consistent periodic waveform by aggregating samples across multiple rotations. The dominant periodic component remains clearly identifiable, demonstrating the robustness of the reconstruction under undersampled conditions.

At high rotational speed (4000 RPM), the sampling density per revolution becomes extremely low, and the raw signal appears highly irregular with no clearly distinguishable peaks. Despite this severe undersampling, the ETS method is still able to recover the dominant periodic structure of the signal. Although some degradation in waveform sharpness and increased noise sensitivity are observed, the reconstructed waveform retains a well-defined peak corresponding to the alignment event.

This behavior highlights the key advantage of the proposed method: even when the raw measurements no longer contain directly detectable peaks, the ETS approach reconstructs a coherent periodic waveform from sparse, nonuniform samples, enabling reliable extraction of the rotation period.

Overall, these results demonstrate that the ETS approach effectively overcomes the limitations imposed by sparse and nonuniform sampling. By exploiting signal periodicity across multiple revolutions, it enables accurate rotational speed estimation across a wide range of operating conditions, including regimes where direct time-domain analysis is not feasible.

### 6.2. Estimation Accuracy for Case 2

The estimation performance for Case 2 is evaluated by comparing the proposed ETS method with the conventional peak-based approach.

To quantitatively assess performance, the normalized root mean square error (NRMSE) and the coefficient of determination ($R^{2}$) are used. The NRMSE is defined as

(21) $$\mathrm{NRMSE}=\frac{\sqrt{\frac{1}{N}\sum_{i=1}^{N}{\left({\hat{RPM}}_{i}-RPM_{i}\right)}^{2}}}{RPM_{\max}-RPM_{\min}},$$

while the coefficient of determination is computed as

(22) $$R^{2}=1-\frac{\sum_{i=1}^{N}{\left({\hat{RPM}}_{i}-RPM_{i}\right)}^{2}}{\sum_{i=1}^{N}{\left(RPM_{i}-\bar{RPM}\right)}^{2}}.$$

Using these metrics, the ETS method for Case 2 yields NRMSE=0.0197 and $R^{2}=0.9964$, indicating excellent agreement between the estimated and reference rotational speeds. For Case 1, the corresponding values are NRMSE=0.0295 and $R^{2}=0.9919$. In contrast, the peak-based method for Case 2 results in NRMSE=0.3886 and $R^{2}=-0.3967$, reflecting poor estimation accuracy under sparse sampling conditions.

These values confirm that the ETS estimates remain very close to the reference measurements over the entire investigated range. In particular, the very low NRMSE indicates that the residual estimation error is small compared with the full speed span, while the $R^{2}$ value close to unity confirms that the proposed method preserves the linear relationship between estimated and reference rotational speeds.

**Figure 7:**
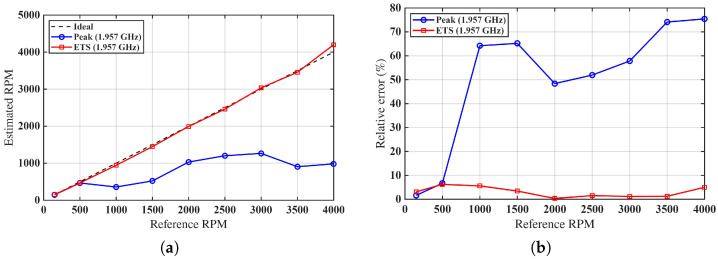
Comparison of peak-based and ETS methods for Case 2 at approximately 1.95 GHz. (**a**) Estimated RPM vs. reference; (**b**) Relative error comparison.

Figure 7 provides a visual comparison of the two methods. At low rotational speeds, both approaches exhibit comparable accuracy. However, as the rotational speed increases beyond approximately 1000 RPM, the peak-based method shows significant deviations from the ideal trend due to missed or partially sampled impedance peaks. In contrast, the ETS method maintains accurate estimation across the full speed range.

This behavior is further highlighted in Figure 7b, where the relative error of the peak-based method exceeds 70% at high rotational speeds, while the ETS method remains below 5%, with near-zero error observed around 2000 RPM. These results confirm that the proposed ETS approach effectively overcomes the limitations of direct peak-based estimation and provides robust performance under severe undersampling conditions.

### 6.3. Discussion

The results demonstrate that the proposed ETS method significantly improves rotational speed estimation under sparse sampling conditions. By exploiting signal periodicity across multiple revolutions, the method overcomes the limitations of peak-based approaches that depend on direct observation of signal extrema [32].

The comparison between Cases 1 and 2 further shows that reconstruction performance depends not only on sampling density but also on the shape and distribution of the impedance variation. Configurations that produce stronger and more distributed responses provide better phase-domain coverage and therefore more accurate estimation. In particular, the results obtained for Case 2 indicate that a shorter interaction interval does not necessarily imply weaker sensing performance. In near-field RF systems, the relevant factor is the strength and spatial distribution of the electromagnetic coupling, which depends on the specific probe–tag geometry and can produce sharper and higher-contrast impedance features that are more favorable for ETS reconstruction.

Although slight degradation is observed at the highest rotational speeds due to reduced sample density and increased noise sensitivity, the ETS method maintains high accuracy across the investigated range.

The experiments were also conducted under varying operating conditions, including different sensing frequencies and two distinct probe–tag alignment configurations. The consistent performance observed across these conditions indicates that the method is not tied to a single narrow operating point, but remains effective under different coupling strengths and sampling regimes. This supports the practical robustness of the proposed ETS framework for RF rotational sensing.

More generally, RF-based sensing is attractive for practical deployments in environments where optical methods may be degraded by dust, occlusion, poor visibility, or line-of-sight limitations. This advantage has been highlighted in previous RF sensing studies, where reliable operation was demonstrated under challenging environmental conditions and non-line-of-sight scenarios [16,33].

**Figure 8:**
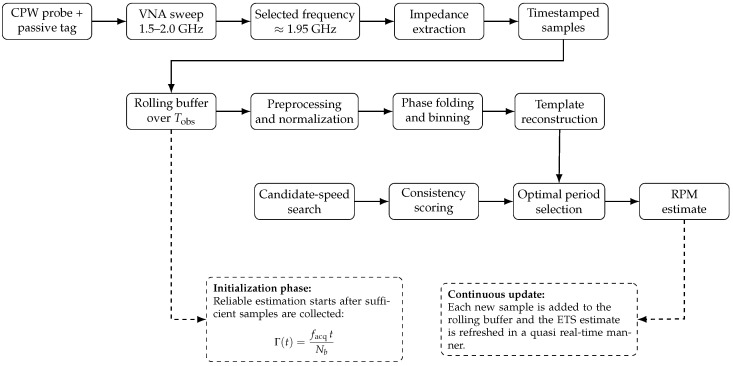
Overall workflow of the proposed RF rotational speed sensing system.

For completeness, the overall workflow of the proposed rotational speed sensing system, from RF measurement to ETS-based speed estimation, is summarized in Figure 8.

## 7. Conclusions

This work presented a rotational speed sensing method based on RF impedance variation using a passive resonant tag and a coplanar waveguide (CPW) probe. The approach exploits the periodic modulation of the real part of the antenna impedance induced by the interaction between the rotating tag and the stationary probe.

To address the limitations of conventional peak-based estimation under sparse sampling conditions, an equivalent-time sampling (ETS) reconstruction method was introduced. By mapping nonuniform impedance samples into an equivalent phase domain, the proposed method reconstructs the periodic waveform associated with a single rotation cycle without requiring direct observation of individual peaks.

Experimental validation over a speed range from 150 RPM to 4000 RPM demonstrated that the proposed method maintains high accuracy even under severe undersampling conditions. While peak-based estimation becomes unreliable above approximately 1000 RPM, the ETS approach achieves relative errors below 5% across the full range, with near-zero error at intermediate speeds. The analysis of different measurement configurations further highlighted the role of signal characteristics in reconstruction performance, showing that stronger and more distributed impedance variations improve phase-domain coverage and estimation accuracy.

Overall, the proposed approach significantly extends the operational range of RF impedance-based rotational sensing without requiring increased acquisition rates. Since the method relies on time-stamped monitoring of the impedance at a selected frequency, it is, in principle, compatible with low-cost RF readers and simplified interrogation circuits. However, this compatibility is inferred from the generality of the signal-processing framework and has not been experimentally validated in this work. Experimental validation was carried out using a vector network analyzer.

In addition, although the proposed ETS framework is computationally lightweight, its effective response time is governed by the observation window required for reliable reconstruction. Therefore, the present implementation should be regarded as a quasi real-time solution, where the achievable latency depends on the tradeoff between update speed and phase-domain coverage. Future work will investigate this tradeoff more systematically, including experimental validation with shorter observation windows and implementation on low-cost RF platforms.

Future work will focus on experimental validation using low-cost RF platforms, improving robustness under noisy conditions, optimizing sampling strategies, and extending the method toward real-time implementation and multi-parameter sensing.

## Figures and Tables

**Table 1:** Comparison of different rotational speed sensing technologies.

Technology	Example Works	Advantages	Limitations
Optical/fiber-optic sensors	[2]	High accuracy; non-contact; immune to electromagnetic interference	Requires line of sight or precise alignment
Magnetic sensors (GMR/Hall)	[3,24]	Robust; low-cost; widely used in industry	Requires magnetic target; affected by electromagnetic interference
Electrostatic sensors	[5]	Contactless operation; simple implementation	Sensitive to noise; limited robustness
SAW sensors	[6]	Passive sensing capability; high sensitivity	Temperature dependence; calibration required
Radar/Doppler sensors	[7,8]	Long-range; non-contact; suitable for harsh environments	High complexity; relatively high cost
RF wireless sensors	[9,10,11]	Wireless operation; passive sensing capability	Limited resolution; sensitivity to alignment and environment
Microwave sensors	[12,13]	Non-contact; high-frequency operation; multifunction sensing	Hardware complexity; calibration requirements
Chipless RFID sensors	[14,15,16,17]	Passive; low cost; no integrated circuits	Limited readout sensitivity; resolution constraints
Encoder/grating-based sensors	[4]	Very high accuracy; industrial standard	Requires mechanical coupling and precise installation
This work	—	Passive RF sensing; no line of sight; compatible with low-cost readers; scalable beyond sampling limits	Requires equivalent-time reconstruction processing

**Table 2:** Experimental data acquisition parameters.

Parameter	Value
Measurement instrument	Anritsu MS46122B Vector Network Analyzer
Measurement mode	Frequency sweep
Measured quantity	Real part of impedance ℜ{Z}
Sweep frequency range	1.5–2.0GHz
Number of sweep points	59
Selected sensing frequency	≈1.95 GHz
Samples per measurement	∼1000
Measurement duration	∼25–30 s
Sampling characteristic	Nonuniform in time
Reference measurement	Optical/laser tachometer

**Table 3:** Comparison of analytical methods for rotational speed estimation.

Method Category	Typical Applications	Strengths	Limitations
Autoregressive (AR) models	Time-series prediction, signal forecasting	Simple implementation; effective for stationary signals	Limited accuracy in fast rotating systems; sensitive to non-stationarity and multipath effects [25]
Lomb–Scargle periodogram	Period detection in unevenly sampled data	Suitable for nonuniform sampling	Assumes sinusoidal signals; not suitable for impulsive peak-based signals [26]
Autocorrelation methods	Periodicity detection, feature extraction	Simple and robust to noise	Requires sufficient samples per cycle; degraded performance under sparse sampling
Epoch folding	Period estimation in cyclic signals	Effective for long periodic observations	Requires multiple cycles and sufficient sampling density
Time–frequency methods (STFT, WVD, Wavelets)	Vibration analysis, fault diagnosis	Good time–frequency localization	Require dense sampling; computationally intensive [27]
Compressive sensing (CS)	Sparse signal reconstruction	Works with limited measurements under sparsity assumptions	Requires prior signal models; high computational complexity [27]
Machine learning approaches	Fault detection, classification	High accuracy with training data	Requires labeled datasets; limited interpretability

**Table 4:** Parameters used in the peak-based rotational speed estimation algorithm.

Parameter	Description	Value
Median filter window	Initial noise suppression	5 samples
Moving-average window	Additional smoothing	5 samples
$\epsilon_{T}$ (initial)	Period tolerance	0.15–0.25
$\epsilon_{T}$ (relaxed)	Relaxed period tolerance	0.30–0.60
$\epsilon_{A}$ (initial)	Amplitude tolerance	0.10–0.15
$\epsilon_{A}$ (relaxed)	Relaxed amplitude tolerance	0.20–0.40
Peak prominence threshold	Minimum prominence for peak detection	max(0.5,0.02ΔZ)

**Table 5:** Approximate number of measurement samples per revolution.

Rotational Speed (RPM)	Samples per Revolution
150	≈14
500	≈4
1000	≈2
2000	≈1
4000	<0.5

**Table 6:** Peak-based rotational speed estimation results for the two measurement configurations at 1.94 GHz.

Nominal RPM	Peak RPM (Case 1)	Error (RPM)	Peak RPM (Case 2)	Error (RPM)
150	149.85	0.15	146.35	3.65
500	309.01	190.99	466.45	33.55
1000	1246.24	246.24	765.48	234.52
1500	733.61	766.39	362.52	1137.48
2000	790.84	1209.16	1032.13	967.87
2500	930.75	1569.25	1201.16	1298.84
3000	1000.40	1999.60	1442.89	1557.11
3500	257.86	3242.14	1346.47	2153.53
4000	635.50	3364.50	982.55	3017.45

**Table 7:** ETS performance at different sensing frequencies.

Frequency (GHz)	Mean Abs. Error (RPM)	Mean Rel. Error (%)	Observation
1.93	51.61	2.37	Lowest average error
1.94	58.50	2.64	Near resonance
1.95	52.29	3.09	More stable at high speeds

**Table 8:** ETS estimation results and performance comparison for the two measurement configurations.

Case	RPM (Ref)	ETS RPM	Abs. Error (RPM)	Rel. Error (%)
Case 1	150	157.93	7.93	5.29
	500	504.12	4.12	0.82
	1000	981.02	18.98	1.90
	1500	1470.20	29.80	1.99
	2000	2092.45	92.45	4.62
	2500	2647.15	147.15	5.89
	3000	3030.93	30.93	1.03
	3500	3548.25	48.25	1.38
	4000	4285.69	285.69	7.14
Case 2	150	154.68	4.68	3.12
	500	503.97	3.97	0.79
	1000	1019.49	19.49	1.95
	1500	1448.26	51.74	3.45
	2000	1948.54	51.46	2.57
	2500	2461.41	38.59	1.54
	3000	2950.57	49.43	1.65
	3500	3455.27	44.73	1.28
	4000	4200.39	200.39	5.01
**Performance Summary**				
**Metric**			**Case 1**	**Case 2**
Mean absolute error (RPM)			73.9	51.6
Mean relative error (%)			3.34	2.37
Worst-case relative error (%)			7.14	5.01

## Data Availability

Data is contained within this article.
